# Dietary Patterns Independent of Fast Food Are Associated with Obesity among Korean Adults: Korea National Health and Nutrition Examination Survey 2010–2014

**DOI:** 10.3390/nu11112740

**Published:** 2019-11-12

**Authors:** Do-Yeon Kim, Ahleum Ahn, Hansongyi Lee, Jaekyung Choi, Hyunjung Lim

**Affiliations:** 1 Research Institute of Medical Nutrition, Kyung Hee University, Seoul 02447, Korea; peacedkim@gmail.com (D.-Y.K.); flowerlhsy@hanmail.net (H.L.); 2 Department of Family Medicine, Research Institute of Medical Science, Konkuk University School of Medicine, Seoul 05030, Korea; arumi81@naver.com; 3 Department of Medical Nutrition, Graduate School of East-West Medical Science, Kyung Hee University, Yong-in 17104, Korea

**Keywords:** adults, dietary pattern, fast food, KNHANES, obesity

## Abstract

Few studies have examined the multifaceted aspects of fast food consumption and dietary patterns for their effects on obesity. We examined the independent associations of obesity with fast food consumption and dietary pattern in Korean adults using a nationally representative cross-sectional survey. A total of 19,017 adults aged 19–64 years participated in the Korea National Health and Nutrition Examination Survey (KNHANES) 2010–2014. Fast food items were removed from diet and then dietary patterns were generated. Multivariate logistic regression analysis was used to examine the odds of overweight/obesity and central obesity according to fast food consumption and dietary patterns. Fast food consumers were about 10% of Korean adults. Both the “White rice and kimchi” pattern and “Meat and alcohol” pattern were associated with low intakes of fiber, calcium, vitamin C, grains, fruit, and milk (*p* < 0.05). Fast food consumers had higher “Meat and alcohol” and “Grains, fruit, and milk” patterns, and they had a lower “White rice and kimchi” pattern than non-fast food-consumers. Fast food consumers were not associated with overweight/obesity, whereas participants with the “Meat and alcohol” pattern had 14% higher overweight/obesity (95% CI: 1.01, 1.28) and 16% higher central obesity (95% CI: 1.00, 1.34). Fast food consumption was not directly associated with obesity, whereas the “Meat and alcohol” pattern had independent associations with overweight/obesity and central obesity among Korean adults.

## 1. Introduction

Obesity is a predisposing factor for several chronic diseases, such as hypertension, type 2 diabetes, cardiovascular disease, and some cancers [1]. The prevalence of obesity has nearly tripled (men: 3.2% to 10.8%, women: 6.4% to 14.9%) worldwide from 1975 to 2014 [2]. In many lower- and middle-income countries in Asia, including South Korea [3], the rate of adult obesity has increased as a negative consequence of increasing economic development, supposedly due to a “nutrition transition.” For example, the traditional dietary pattern high in grains, vegetables, and oilseeds is being replaced by a Western pattern high in fats, caloric sweeteners, and animal-sourced foods [4,5]. In line with that trend, fast food consumption doubled in South Korea from 1998 to 2009 [6]. 

Several researchers are concerned about the increasing consumption of fast food [7], which is characterized by high energy density [8], excessive portion sizes, high glycemic load [9], and low intakes of vegetables, fruits, and milk [10]. Some studies suggest an association between fast food intake and poor health outcomes [9,11,12]. On the other hand, others reported that are no significant associations between fast food intake and health outcomes, such as obesity [7,13,14]. These inconsistent results may be because other factors that correlate with fast food consumption have been limited to assess dietary factors, such as access to fast food restaurants [15], dietary preferences, and poor eating behaviors [16], including dietary patterns, rather than fast food consumption itself [10,14]. 

A study tested the independent association of obesity with dietary patterns and fast food items by collecting detailed food intake data among children in the US [10], but to our knowledge, no study exists for adults. In Asian countries, many studies have been conducted to investigate the association between dietary pattern and obesity [5,17,18], however, no study has examined for an independent association of weight gain with dietary pattern and fast food consumption.

To fill this gap, we examine the independent associations of overweight/obesity with dietary pattern and fast food consumption in Korean adults using nationally representative cross-sectional survey data. 

## 2. Materials and Methods

### 2.1. Participants and Exclusion Criteria

We used data from the fifth and sixth Korea National Health and Nutrition Examination Survey (KNHANES) in 2010 and 2014. The KNHANES is a nationally representative, cross-sectional survey of the South Korean population that uses a clustered, multistage, stratified, and rolling sampling design to represent non-institutionalized civilian Koreans [19]. The KNHANES V–VI protocols were approved by the Institutional Review Board of the Korean Center for Disease Control and Prevention (IRB No. 2010–02CON–21–C, 2011–02CON–06–C, 2012–01EXP–01–2C, 2013–07CON–03–4C, 2013–12EXP–03–5C).

We merged the 2010–2014 KNHANES data on a total of 41,102 participants and then sequentially excluded participants with extreme energy intake (<500 kcal/day or >5000 kcal/day) (*n* = 4744) [20]; who were pregnant (*n* = 133); who were <19 years or ≥65 years (*n* = 15,824); who were missing height or weight measurements (*n* = 62); or who had missing weight values (*n* = 1322). After applying these exclusion criteria, the total number of participants for this analysis was 19,017 (7516 men and 11,563 women). 

### 2.2. Anthropometric Measurements 

Height (Seca 225; SECA, Hamburg, Germany), weight (GL–6000–20; CASKOREA Co., Ltd., Seoul, Korea), and waist circumference (Seca 200; SECA, Hamburg, Germany) were obtained using standardized techniques and calibrated equipment. Body mass index (BMI) was calculated from these measurements as weight (kg) divided by height squared (m^2^). Overweight (BMI of 23–25 kg/m^2^) and obesity (BMI ≥ 25 kg/m^2^) were defined based on World Health Organization recommendations for Asians [21].

### 2.3. Dietary Assessment and Fast Food Consumption 

Dietary data were assessed by trained dietary interviewers using the single 24-h recall method in the homes of the participants one week after the health interview and health examination. Energy and nutrient intakes for each subject were calculated using the Korean Foods and Nutrients Database of the Rural Development Administration [22,23], which contains 3095 food items. 

Fast food consumption was defined as the intake of any of 48 Western fast food items (e.g., hamburgers, French fries, pizza, onion rings, fried chicken, sandwiches, apple pie, and cheese sticks). We classified adults as fast food consumers (FF consumers) if they consumed at least one food sourced from fast food on their day’s recall and non-fast food consumers (non-consumers) if they did not consume any fast food. 

### 2.4. Food Grouping 

We divided food data into 25 food groups from the 18 food groups in the Korean Nutrient Database [24] to simplify the interpretation of components. Intake of grains and their products is very high in the Korean population; therefore, we subdivided this food group into four subgroups based on the nutrient profile: white rice, other grains, flour and bread, and noodles. Kimchi (traditional fermented cabbage) was also placed in a group separate from the vegetable group because it is a traditional side dish in Korea. The beverage group was disaggregated into soda, sugar-sweetened beverages (SSBs), and non-sugar beverages based on sugar content [25]. 

### 2.5. Dietary Pattern Analysis Excluding Fast Food

To identify dietary patterns excluding fast food, all fast food items were removed from the 25 food groups. Because of the non-normal distribution of data, we transformed the percentage of total energy contribution for each food group per day and then dietary patterns were generated by a K-means cluster analysis using SAS FASTCLUS (SAS Institute, Cary, NC, USA). This K-means procedure generates clusters by comparing the Euclidean distance between each subject and each cluster center [26]. Two to five solutions were examined to assess which set of clusters were more meaningful in determining dietary patterns. In the end, three clusters were identified as distinct patterns and labeled with descriptive names.

Almost half of the subjects (48%) were assigned to the “White rice and kimchi” dietary pattern, in which consumption of white rice accounted for about 50% of total energy intake, and consumption of kimchi was higher than in the other two groups. A total of 40% of the subjects were assigned to the Grain, fruit, and milk dietary pattern, which was characterized by a variety of food groups, such as whole grains, bread, noodles, potatoes, nuts, fruit, eggs, milk, and oils. The remaining 12% of subjects were assigned to the “Meat and alcohol” dietary pattern based on their predominant food groups (Supplementary Table S1). 

### 2.6. Covariates

Information on socio-demographic characteristics and health-related behaviors were obtained using in-person interviews by a trained researcher. Household income was divided into quartiles. Education level was categorized into three groups: ≤middle school, high school, and ≥college degree. Region was divided into two groups: urban and rural. Smoking status was divided into three groups: non-smokers, former smokers, and current smokers. Physical activity was assessed using the short form of International Physical Activity Questionnaire (IPAQ) [27]. The IPAQ suggests the level of physical activity by a Metabolic Equivalent Task (MET): vigorous MET-minutes/week = 8.0 × minutes of activity per day × days of activity per week, moderate MET-minutes/week = 4.0 × minutes of activity per day × days of activity per week, and walking MET-minutes/week = 3.3 × minutes of activity per day × days of activity per week. Then, physical activity was divided into three groups: “Health-Enhancing” when the total score was 3000 MET-minutes/week or more, “Active” when the total score was 600 MET-minutes or more, and “Inactive” when the total score was below 600 MET-minutes [27]. Total energy intake was divided into age and sex-specific quintiles. Weight status was categorized into three groups: non-overweight, overweight, and obese. 

### 2.7. Statistical Analysis

The analysis was performed using PROC SURVEY in SAS 9.4 (SAS Institute, Cary, NC, USA) to take into account the complex sampling design and appropriate sampling weights for the national survey. Two-sided chi-square and *t*-tests (*p* < 0.05) were used to compare sociodemographic characteristics according to fast food consumption and dietary pattern. We tested the associations between dietary pattern and dietary outcomes using the “Grain, fruit, and milk” pattern as reference. To estimate the associations between fast food consumption and each dietary pattern, we used multiple binary logistic regression models using the non-consumer as a reference. We adjusted for age, sex, household income, education, region, smoking, physical activity, total energy intake, and weight status.

Then, we used multivariate-adjusted logistic regression models to estimate the odds of overweight/obesity for fast food consumption and each dietary pattern after adjusting for age, sex, household income, education, total energy intake, smoking, and physical activity (model 1). Model 2 was adjusted for potential confounders and dietary pattern to determine the direct association between fast food consumption and weight status, and adjusted for confounders with fast food consumption to estimate the independent association between dietary pattern and weight status. Statistical significance was achieved when *p* < 0.05.

## 3. Results

### 3.1. General Characteristics

General characteristics according to fast food consumption and dietary pattern are shown in Table 1. A total of 9.7% of adults were categorized into FF consumers. In general, FF consumers of all dietary patterns tended to be younger and more highly educated than non-consumers (*p* < 0.0001). According to dietary pattern, followers of the “Grain, fruit, eggs, and milk” pattern had a higher proportion of young, female, and highly educated individuals and fewer smokers regardless of fast food consumption (*p* < 0.0001). Those following the “White rice and kimchi pattern” were more likely to be older with low education and those following the “Meat and alcohol pattern” were more likely to be male and current smokers regardless of fast food consumption (*p* < 0.0001). 

### 3.2. Total Intake and Dietary Intake Excluding Fast Food

The findings for total intake and dietary intake excluding fast food according to fast food consumption are shown in Table 2. A greater intake of meat and its products and soda and a lower intake of grain, legumes, nuts and seeds, vegetables, and fruit were observed among FF consumers not only for total intake (10.6 ± 0.2, 2.4 ± 0.1, 3.3 ± 0.2, 1.4 ± 0.1, 0.6 ± 0.1, 2.4 ± 0.1, and 2.9 ± 0.1 kcal/day, respectively) but also for dietary intake excluding fast food (12.9 ± 0.3, 3.3 ± 0.2, 4.0 ± 0.2, 1.7 ± 0.1, 0.8 ± 0.1, 2.8 ± 0.1, and 3.4 ± 0.1 kcal/day, respectively) than non-consumers (*p* < 0.05). In addition, FF consumers had higher total energy (2741 ± 30 kcal/day) and fat intake (25.3 ± 0.2%) than non-consumers (2048 ± 9 kcal/day and 18.7 ± 0.1%, respectively; *p* < 0.05) in total intakes, and they also had higher total energy and fat intake even in dietary intake excluding fast food (2230 ± 26 kcal/day and 22.5 ± 0.2%, respectively) than non-consumers (2048 ± 9 kcal/day and 18.7 ± 0.1%, respectively; *p* < 0.05). 

### 3.3. Dietary Intakes According to Dietary Pattern 

A multivariate regression model of each dietary intake with dietary pattern is shown in Table 3. The “White rice and kimchi” pattern and the “Meat and alcohol” pattern were associated with a lower consumption of healthy food and nutrient values than the “Grain, fruit, and milk” pattern. Participants following the “White rice and kimchi” pattern consumed significantly lower amounts of grains (β = −60.5; 95% CI: −68.9, −52.1 g), milk and dairy products (β = −42.3; 95% CI: −47.5, −37.2 g), fruit (β = −33.0; 95% CI: −38.2, −27.7 g), oils (β = −10.3; 95% CI: −13.4, −7.1 g), nuts and seeds (β = −8.9; 95% CI: −11.8, −6.0 g), and protein (β = −5.4; 95% CI: −6.4, −4.3 g), fiber (β = −0.6; 95% CI: −0.8, −0.4 g), calcium (β = −51.4; 95% CI: −62.7, −40.1 g), and vitamin C (β = −11.1; 95% CI: −15.2, −7.0 g) than those following the “Grain, fruit, and milk” pattern. For those following the “Meat and alcohol” pattern, the consumption of grains (β = −86.3; 95% CI: −96.1, −76.5 g), fruit (β = −62.7; 95% CI: −70.4, −54.9 g), milk and dairy products (β = −59.1; 95% CI: −65.7, −52.4 g), legumes (β = −18.0; 95% CI: −22.7, −13.2 g), nuts and seeds (β = −6.3; 95% CI: −10.4, −2.1 g), eggs (β = −11.8; 95% CI: −15.6, −8.0 g), oils (β = −11.3; 95% CI: −16.7, −5.8 g), and fiber (β = −1.7; 95% CI: −2.0, −1.4 g), calcium (β = −113.1; 95% CI: −130.5, −95.7 mg), vitamin A (β = −96.3; 95% CI: −167.2, −25.4 μgRE), and vitamin C (β = −33.2; 95% CI: −39.7, −26.6 mg) was lower, although total energy intake (β = 171.3; 95% CI: 137.8, 204.7 kcal) was higher than the “Grain, fruit, and milk” pattern.

### 3.4. Dietary Pattern According to Fast Food Consumption

Adjusted OR and 95% CI for the dietary pattern according to fast food consumption are shown in Table 4. The adjusted OR following the “Meat and alcohol” pattern increased by 1.2-fold (OR: 1.21; 95% CI: 1.02, 1.43) and following the “Grain, fruit, and milk” pattern increased by 2-fold (OR: 2.00; 95% CI: 1.76, 2.27) among FF consumer compared with non-consumers after adjusting for potential confounders. On the other hand, FF consumers were 59% less likely than non-consumers to follow the “White rice and kimchi” pattern (OR: 0.41; 95% CI: 0.35, 0.47). 

### 3.5. Overweight/obesity or Central Obesity According to Fast Food Consumption and Dietary Pattern

Adjusted OR and 95% CI for overweight/obesity or central obesity according to fast food consumption and dietary pattern are shown in Table 5. Although fast food consumption was not associated with overweight/obesity or central obesity after adjusting for covariates and dietary patterns, those who followed the “Meat and alcohol” pattern had a 14% higher prevalence of overweight/obesity (OR: 1.14; 95% CI: 1.04, 1.28) and a 16% higher prevalence of central obesity (OR: 1.16; 95% CI: 1.00, 1.34) than those who followed the “Grain, fruit, and milk” pattern after adjusting for covariates and fast food consumption. Furthermore, decreased odds of overweight/obesity were associated with the “White rice and kimchi” pattern (OR: 0.88; 95% CI: 0.81, 0.95), although central obesity was not associated after controlling for covariates and fast food consumption. 

## 4. Discussion 

This study contributes to the growing literature on unhealthy eating and overweight/obesity and is, to our knowledge, the first to examine independent associations among dietary patterns, fast food consumption, and overweight/obesity within a nationally representative sample of Korean adults. We found that the dietary pattern associated with health outcomes when fast food consumption is controlled, in particular the “Meat and alcohol” pattern, and that fast food consumption was not associated with weight gain when dietary patterns and other covariates were controlled. 

Obesity results from a complex interaction of gender, environmental, genetic, and behavioral factors, and existing disease [28]. Among the behavioral factors, diet plays a major role in the development of overweight/obesity [4]. Previous research showed that people who consume fast food have higher energy and fat intakes, which are important contributors to overweight/obesity, suggesting that regulating the consumption of fast food might improve diets and health [14,15,29]. Our results also noted that FF consumers had higher total energy and fat intakes than non-consumers even when fast food items were excluded in the diet. Although the proportion of participants who consumed FF was low (10%), but those were doubled over an 11-year period [6], and several Asian researchers are concerned about the increasing consumption of fast food because it has been suggested to be linked to obesity in Asia [7,13]. However, emerging evidence suggests that obesity is associated with an individual’s dietary preferences and poor eating behavior, and sociodemographic characteristics, rather than being directly related to fast food [6,16]. In the crude model without adjustment in our results, fast food consumption was positively associated with overweight/obesity and central obesity (data not shown), but the significance disappeared after adjustment for potential confounders, which provides important information about the direct factors and prevalence of overweight/obesity. 

Our results are of international significance because they contribute to an emerging evidence base that suggests that dietary pattern is independently associated with overweight/obesity regardless of fast food consumption [10], which was consistent with the result of subgroup analysis on non-consumer (data not shown). Furthermore, when we separated eating behavior into fast food consumption and dietary patterns with the fast food items removed, we were able to compare the probability of overweight/obesity for different eating behaviors at the same time. Our results are unique in showing that the “Meat and alcohol” pattern was positively associated with overweight/obesity, whereas the “White rice and kimchi” pattern showed an opposite association. Previous studies suggested that the consumption of red and processed meats was associated with the risk of obesity [30]. With respect to “White rice and kimchi” dietary pattern, a representative Korean study reported that a carbohydrate intake that composes more than 60% of total energy intake and the “White rice and kimchi” pattern had a lowering effect on BMI due to lower intakes of total fat. However, lowering HDL-cholesterol and increasing serum triglycerides are negative effects of the “White rice and kimchi” pattern; thus, even though that pattern is not positively associated with obesity, it does contribute to high intakes of carbohydrate and sodium and provides limited food composition [31]. In agreement, we found that people following the “White rice and kimchi” pattern derived 69% of their energy intake from carbohydrates (data not shown) and it may influence the lower prevalence of overweight/obesity. 

In light of our findings, we suggest that dietary patterns are a more crucial factor in overweight/obesity than fast food consumption. Therefore, research is needed to determine what dietary patterns are healthy while excluding fast food consumption. Combining previous evidence that healthy patterns are characterized by high intakes of whole grains, fruits, and legumes and are rich in nutrients, such as antioxidants and associated with a reduction in metabolic risk factors [31,32], our results suggest that the “Grains, fruit, and milk” pattern could have preventive effects on weight gain through the combined influence of nutrients and food components compared to the “Meat and alcohol” pattern. Furthermore, for the first time, to our knowledge, we showed that adult Asian FF consumers were linked to the “Meat and alcohol” pattern or “Grain, fruit, and milk” pattern. Previous studies showed that FF consumers were associated with a Western dietary pattern but those analyses were based on US children [10]. Previous studies and our results thus suggest that those who consume the healthy “Grain, fruit, and milk” pattern are less likely to be overweight/obese than those who eat the “Meat and alcohol” pattern regardless of fast food consumption. 

Our study has several limitations. First, dietary intake was estimated based on a 24-h dietary recall, which might not have been representative of a subject’s typical intake. However, at the population level, it can provide rich details about mean dietary intake for a given day [33]. Second, this was a cross-sectional study; therefore, our study precludes inferences of causation, and the possibility of reverse causation remains. 

Despite these limitations, the present study has many strengths. First, we used data from a nationally representative sample and took into account the complex sampling design effects to provide representative estimates. Second, we sufficiently analyzed a considerable range of available fast food items to identify FF consumers, and we separated fast food items from the rest of their diet. Third, most previous studies have considered only dietary patterns or fast food consumption, but we investigated the participants’ dietary patterns separate from the fast food items they ate and used fast food consumption to identify dietary factors associated with obesity in adults, which allowed us to find that fast food might be not solely responsible for obesity.

## 5. Conclusions

In conclusion, we found that fast food consumption was not directly associated with obesity, whereas the “Meat and alcohol” pattern had independent associations with overweight/obesity and central obesity independent of fast food. Even if fast food is high in calories, the unhealthy dietary pattern has a stronger correlation with overweight/obesity and central obesity than fast food consumption on its own. Our results indicate that more attention should be paid to dietary patterns to prevent and manage overweight/obesity and central obesity among adults in South Korea. 

## Figures and Tables

**Table 1:** General characteristics according to fast food consumption and dietary patterns in adults when fast food items are excluded: KNHANES 2010–2014 ^1^.

		Grains, Fruit, and Milk Pattern ^2^					White Rice and Kimchi Pattern					Meat and Alcohol Pattern						
		Non-Consumers ^3^		FF Consumers			Non-Consumers		FF Consumers			Non-Consumers		FF Consumers				
		(*n* = 6541; 84.3%)		(*n* = 1085; 15.7%)		*p*-Value ^4^	(*n* = 8695; 94.5%)		(*n* = 454; 5.5%)		*p*-Value ^4^	(*n* = 1937; 85.7%)		(*n* = 305; 14.3%)		*p*-Value ^4^	*p*-Value ^5^	*p*-Value ^6^
Age						<0.0001					<0.0001					<0.0001	<0.0001	<0.0001
	19–39 years	48.0	(0.8)	72.6	(1.6)		37.9	(0.8)	59.6	(2.7)		43.0	(1.4)	71.7	(2.9)			
	40–64 years	52.0	(0.8)	27.4	(1.6)		62.1	(0.8)	40.4	(2.7)		57.0	(1.4)	28.3	(2.9)			
Sex						0.0546					0.7924					0.9789	<0.0001	<0.0001
	Male	40.3	(0.7)	44.0	(1.7)		51.9	(0.6)	52.6	(2.7)		73.9	(1.0)	73.8	(2.6)			
	Female	59.7	(0.7)	56.0	(1.7)		48.1	(0.6)	47.4	(2.7)		26.1	(1.0)	26.2	(2.6)			
Education						<0.0001					<0.0001					<0.0001	<0.0001	<0.0001
	≤Middle school	12.9	(0.6)	3.5	(0.5)		23.6	(0.7)	12.0	(1.7)		13.7	(0.9)	5.2	(1.4)			
	High school	30.7	(0.8)	27.2	(1.6)		34.4	(0.7)	26.5	(2.6)		35.3	(1.4)	27.9	(3.1)			
	≥College degree	56.4	(0.9)	69.2	(1.6)		42.0	(0.8)	61.5	(3.0)		51.1	(1.5)	66.9	(3.1)			
Household income						0.3162					0.2808					0.2902	<0.0001	0.0554
	Low (Q1)	7.5	(0.5)	9.3	(1.3)		11.9	(0.5)	9.4	(1.9)		8.3	(0.8)	7.5	(1.7)			
	Low middle (Q2)	24.5	(0.8)	22.5	(1.7)		29.2	(0.7)	27.4	(2.6)		25.7	(1.3)	28.1	(3.1)			
	Middle-High (Q3)	32.0	(0.8)	32.9	(1.9)		31.1	(0.7)	36.1	(2.8)		32.9	(1.3)	27.1	(3.0)			
	High (Q4)	36.1	(1.0)	35.2	(1.9)		27.8	(0.8)	27.1	(2.6)		33.1	(1.4)	37.3	(3.3)			
Region						0.1189					0.0539					0.0240	<0.0001	0.1463
	Urban	76.6	(1.1)	79.4	(1.9)		68.4	(1.1)	73.9	(2.7)		71.9	(1.6)	79.2	(2.9)			
	Rural	23.4	(1.1)	20.6	(1.9)		31.6	(1.1)	26.1	(2.7)		28.1	(1.6)	20.8	(2.9)			
Smoking						0.3475					0.5807					0.6760	<0.0001	<0.0001
	Current	20.1	(0.7)	22.3	(1.5)		25.9	(0.6)	24.1	(2.5)		45.8	(1.4)	48.7	(3.4)			
	Past	16.5	(0.6)	17.0	(1.5)		19.1	(0.5)	17.7	(2.3)		24.0	(1.2)	21.8	(2.7)			
	Never	63.4	(0.7)	60.7	(1.9)		55.1	(0.6)	58.2	(2.9)		30.1	(1.2)	29.5	(2.9)			
Physical activity																		
	Inactive	35.5	(0.8)	36.7	(1.8)	0.4850	39.8	(0.7)	39.7	(2.7)	0.9953	33.2	(1.3)	34.3	(3.2)	0.1332	<0.0001	0.7707
	Active	44.3	(0.8)	42.0	(1.8)		39.5	(0.6)	39.8	(2.7)		39.1	(1.4)	44.1	(3.3)			
	Health enhancing	20.1	(0.6)	21.3	(1.5)		20.7	(0.6)	20.6	(2.4)		27.7	(1.2)	21.6	(2.5)			

KNHANES, Korea national health and nutrition examination survey; FF, fast food; non-consumer, non-fast food consumers. ^1^ Values are % (SE). Data were weighted to represent adults aged 19–64 years from KNHANES 2010–2014. ^2^ Dietary patterns excluding fast food were determined by cluster analysis using dietary intake (kcal/day) minus any fast food items consumed. ^3^ Fast food consumers are defined as those who consumed more than one fast food item, and non-consumers are defined as those who did not consume any fast food. ^4^ *p*-values for differences between FF consumers and non-consumers in each cluster using chi-square test for proportions and analysis of variance for means. ^5^ *p*-values for differences across clusters in non-consumers using chi-square test for proportions and analysis of variance for means. ^6^ *p*-values for differences across clusters in FF consumers using chi-square test for proportions and analysis of variance for means.

**Table 2:** Total dietary intake and dietary intake excluding fast food items according to fast food consumption among adults: KNHANES 2010–2014 ^1^.

	Total Intake		Intake Minus FF ^2^	
	Non-Consumer ^3^	FF Consumer	Non-Consumer	FF Consumer
	(*n* = 17,173; 90.3%)	(*n* = 1844; 9.7%)	(*n* = 17,173; 90.3%)	(*n* = 1844; 9.7%)
Food group (% of energy) ^4^				
White rice	31.90 ± 0.21	17.91 ± 0.37 *	31.90 ± 0.21	21.56 ± 0.42 *
Grains	6.06 ± 0.10	3.34 ± 0.15 *	6.06 ± 0.10	3.98 ± 0.18 *
Sweets	1.89 ± 0.03	1.57 ± 0.07 *	1.89 ± 0.03	1.90 ± 0.09
Legumes	2.36 ± 0.04	1.43 ± 0.07 *	2.36 ± 0.04	1.73 ± 0.09 *
Nuts and seeds	1.02 ± 0.03	0.64 ± 0.06 *	1.02 ± 0.03	0.76 ± 0.07 *
Vegetables	3.16 ± 0.03	2.36 ± 0.08 *	3.16 ± 0.03	2.84 ± 0.09 *
Kimchi	1.43 ± 0.02	0.81 ± 0.03 *	1.43 ± 0.02	0.98 ± 0.03 *
Fruit	4.45 ± 0.08	2.89 ± 0.12 *	4.45 ± 0.08	3.44 ± 0.14 *
Meat and its products	8.81 ± 0.11	10.63 ± 0.23 *	8.81 ± 0.11	12.93 ± 0.28 *
Eggs	1.90 ± 0.03	1.69 ± 0.06 *	1.90 ± 0.03	2.04 ± 0.08
Fish and shellfish	3.71 ± 0.05	2.41 ± 0.10 *	3.71 ± 0.05	2.91 ± 0.13 *
Milk and dairy products	3.53 ± 0.07	3.46 ± 0.14	3.53 ± 0.07	4.42 ± 0.21 *
Oils	3.27 ± 0.03	4.50 ± 0.10 *	3.27 ± 0.03	5.49 ± 0.13 *
Soda	0.56 ± 0.02	2.40 ± 0.11 *	0.56 ± 0.02	3.30 ± 0.16 *
SSBs	3.11 ± 0.05	2.44 ± 0.11 *	3.11 ± 0.05	3.00 ± 0.15
Alcohol	4.22 ± 0.11	4.30 ± 0.25	4.22 ± 0.11	5.02 ± 0.29 *
Total energy, kcal/day	2048.0 ± 8.9	2740.6 ± 30.2 *	2048.0 ± 8.9	2229.9 ± 26.3 *
Total carbohydrate, % of energy	63.6 ± 0.2	52.5 ± 0.3 *	63.6 ± 0.2	56.6 ± 0.4 *
Total fat, % of energy	18.7 ± 0.1	25.3 ± 0.2 *	18.7 ± 0.1	22.5 ± 0.2 *

KNHANES, Korea national health and nutrition examination Survey; FF, fast food; non-consumer, non-fast food consumers. ^1^ Values are mean ± SE. Data were weighted to represent adults aged 19–64 years from KNHANES 2010–2014. * Statistically different from fast food non-consumers (*p* < 0.05 with *t*-test). ^2^ Dietary intake minus fast food items. ^3^ Fast food consumers are defined as those who consumed more than one fast food item, and non-consumers are defined as those who did not consume any fast food. ^4^ Percentage of total energy intake from each food group in total dietary intake and dietary intake minus fast food according to fast food consumption.

**Table 3:** Multiple linear regression analysis of the independent associations between dietary pattern minus fast food and dietary outcomes among adults: KNHANES 2010–2014 ^1^.

		Dietary Pattern ^2^	
		White Rice and Kimchi Pattern	Meat and Alcohol Pattern
Food groups (kcal/day)			
	White rice	**505.4 (494.8, 516.1)**	**36.8 (21.4, 52.2)**
	Grains	**−60.5 (−68.9, −52.1)**	**−86.3 (−96.1, −76.5)**
	Legumes	−3.2 (−6.6, 0.3)	**−18.0 (−22.7, −13.2)**
	Nuts and seeds	**−8.9 (−11.8, −6.0)**	**−6.3 (−10.4, −2.1)**
	Vegetables	−1.0 (−3.0, 1.0)	**4.9 (1.7, 8.2)**
	Kimchi	**5.0 (3.8, 6.1)**	−1.0 (−2.7, 0.6)
	Fruit	**−33.0 (−38.2, −27.7)**	**−62.7 (−70.4, −54.9)**
	Meat and its products	**−44.9 (−53.2, −36.6)**	**232.6 (207.1, 258.1)**
	Eggs	−1.5 (−3.8, 0.9)	**−11.8 (−15.6, −8.0)**
	Fish and shellfish	**9.1 (4.5, 13.6)**	**18.6 (10.1, 27.2)**
	Milk and dairy products	**−42.3 (−47.5, −37.2)**	**−59.1 (−65.7, −52.4)**
	Oils	**−10.3 (−13.4, −7.1)**	**−11.3 (−16.7, −5.8)**
	Soda	**−9.5 (−11.9, −7.1)**	**−12.4 (−16.4, −8.4)**
	SSBs	−0.7 (−4.3, 3.0)	**−18.2 (−23.6, −12.7)**
	Alcohol	−0.9 (−5.0, 3.2)	**594.7 (572.2, 617.2)**
Nutrients			
	Total energy (kcal/day)	**−62.8 (−78.4, −47.1)**	**171.3 (137.8, 204.7)**
	Protein (g/day)	**−5.4 (−6.4, −4.3)**	**8.3 (6.0, 10.6)**
	Fat (g/day)	**−13.0 (−13.9, −12.1)**	0.6 (−1.5, 2.8)
	Carbohydrate (g/day)	**15.5 (12.1, 18.8)**	**−102.7 (−107.9, −97.4)**
	Fiber (g/day)	**−0.6 (−0.8, −0.4)**	**−1.7 (−2.0, −1.4)**
	Calcium (mg/day)	**−51.4 (−62.7, −40.1)**	**−113.1 (−130.5, −95.7)**
	Vitamin A (μgRE/day)	−15.8 (−58.9, 27.2)	**−96.3 (−167.2, −25.4)**
	Vitamin C (mg/day)	**−11.1 (−15.2, −7.0)**	**−33.2 (−39.7, −26.6)**

KNHANES, Korea national health and nutrition examination survey; FF, fast food; non-consumer, non-fast food consumers. ^1^ Data were weighted to represent adults aged 19–64 years from KNHANES 2010–2014. Values are β coefficients (95% CIs) of dietary outcomes (food groups and nutrients), with dietary pattern as the independent variable using the “Grain, fruit, and milk” pattern as a reference. The model was adjusted for age, sex, income, education, region, smoking, total energy intake, and weight status. Bold indicates significance at *p*-value < 0.05. ^2^ Dietary patterns minus fast food were determined by cluster analysis using dietary intake (kcal/day) excluding any fast food items consumed.

**Table 4:** ORs (95% CIs) for dietary pattern minus fast food items by fast food consumption among adults: KNHANES 2010–2014 ^1^.

	Fast Food Consumption ^2^	
	Non-Consumer	FF Consumer
	(*n* = 17,173; 90.3%)	(*n* = 1844; 9.7%)
Fast food intake (% of energy) ^3^	0	13.27 (0.03, 84.13)
Multivariable-adjusted OR		
Grain, fruit, and milk pattern		
Model 1 ^4^	1.0 (reference)	**2.08 (1.83, 2.36)**
Model 2	1.0	**2.00 (1.76, 2.27)**
White rice and kimchi pattern		
Model 1	1.0	**0.37 (0.32, 0.42)**
Model 2	1.0	**0.41 (0.35, 0.47)**
Meat and alcohol pattern		
Model 1	1.0	**1.45 (1.23, 1.71)**
Model 2	1.0	**1.21 (1.02, 1.43)**

KNHANES, Korea national health and nutrition examination Survey; FF, fast food; non-consumer, non-fast food consumers. ^1^ Data were weighted to represent adults aged 19–64 years from KNHANES 2010–2014. Dietary patterns minus fast food were determined by cluster analysis using intake (kcal/day) and excluding any fast food items consumed. ^2^ Fast food consumers are defined as those who consumed more than one fast food item, and non-consumers are defined as those who did not consume any fast food. ^3^ Values are medians (ranges). ^4^ ORs (95% CIs) for dietary pattern minus any fast food consumed by fast food consumption using multiple binary logistic regression models. Model 1 was adjusted for age, sex, income, education, region, physical activity, and smoking. Model 2 was adjusted for Model 1 plus total energy intake and weight status. Bold indicates significance at *p*-value < 0.05.

**Table 5:** ORs (95% CIs) for weight status with fast food consumption and dietary pattern minus any fast food items consumed among adults: KNHNES 2010–2014 ^1^.

		FF Consumption ^2^	Dietary Pattern ^3^	
			White Rice and Kimchi	Meat and Alcohol
Overweight/obesity				
	Model 1 ^4^	1.04 (0.92, 1.17)	**0.88 (0.82, 0.96)**	**1.14 (1.01, 1.28)**
	Model 2	1.07 (0.94, 1.20)	**0.88 (0.81, 0.95)**	**1.14 (1.01, 1.28)**
Central obesity				
	Model 1	1.15 (0.97, 1.36)	1.09 (0.99, 1.20)	**1.16 (1.01, 1.35)**
	Model 2	1.14 (0.96, 1.34)	1.08 (0.98, 1.19)	**1.16 (1.00, 1.34)**

KNHANES, Korea national health and nutrition examination Survey; FF, fast food; non-consumer, non-fast food-consumers. ^1^ Data were weighted to represent adults aged 19–64 years from KNHANES 2010–2014. ^2^ Fast food consumers are defined as those who consumed more than one fast food item, and non-consumers are defined as those who did not consume any fast food. ^3^ Dietary patterns minus fast food were determined by cluster analysis using intake (kcal/day) and excluding any fast food items consumed. ^4^ ORs (95% CIs) for weight status (overweight/obesity and central obesity) with fast food consumption and dietary pattern minus fast food (reference: “Grain, fruit, and milk” pattern). Model 1 was adjusted for age, sex, income, education, region, smoking, total energy intake, and physical activity. Model 2 was adjusted for dietary pattern excluding fast food to find associations with fast food consumption and was adjusted for fast food consumption to find associations with dietary patterns excluding fast food. Bold indicates significance at *p*-value < 0.05.

## Supplementary Materials

The following are available online at https://www.mdpi.com/2072-6643/11/11/2740/s1, Table S1. Mean of food group intake according to dietary pattern minus any fast food items consumed among adults: KNHNES 10–14.

## Abbreviation:

KNHANES	Korea National Health and Nutrition Examination Survey
SSBs	Sugar-sweetened beverages
BMI	Body mass index
FF	Fast foo
OR	Odds ratio
CI	Confidence interval
