# Supplementary material for: Dietary Patterns Independent of Fast Food Are Associated with Obesity among Korean Adults: Korea National Health and Nutrition Examination Survey 2010–2014
Source: Nutrients. 2019 Nov 12;11(11):2740. doi: 10.3390/nu11112740 (PMC6893752; doi:10.3390/nu11112740)
Supplement: Supplementary file 1 [file nutrients-11-02740-s001.pdf]

Supplementary Table S1. Mean of food group intake according to dietary pattern minus any fast food items consumed among adults: KNHNES 10–14[1]

Food group (% energy)	Grain, fruit, & milk pattern[2] (n=7626)		White rice & kimchi pattern (n=9149)		Meat & alcohol pattern (n=2242)		
	Mean	SE	Mean	SE	Mean	SE	p-value[3]
White rice	16.66	0.14 [c]	**46.16**	**0.18** [a]	19.46	0.23 [b]	<.0001
Grains	**7.64**	**0.18** [a]	5.16	0.09 [b]	2.91	0.11 [c]	<.0001
Flour and bread	**13.36**	**0.23** [a]	4.22	0.09 [b]	3.05	0.12 [c]	<.0001
Noodle	**10.17**	**0.22** [a]	2.60	0.10 [c]	4.01	0.19 [b]	<.0001
Potatoes	**2.66**	**0.09** [a]	1.78	0.05 [b]	1.27	0.07 [c]	<.0001
Sweets	**2.23**	**0.06** [a]	1.73	0.03 [b]	1.46	0.05 [c]	<.0001
Legumes	2.42	0.06 [a]	2.42	0.05 [a]	1.46	0.06 [b]	<.0001
Nuts and seeds	**1.26**	**0.06** [a]	0.81	0.03 [b]	0.85	0.05 [b]	<.0001
Vegetables	3.12	0.04 [a]	3.19	0.03 [a]	2.93	0.05 [b]	<.0001
Kimchi	1.19	0.02 [b]	**1.65**	**0.02** [a]	1.08	0.03 [c]	<.0001
Mushrooms	0.10	0.01 [a]	0.10	0.01 [ab]	0.08	0.01 [b]	0.0228
Fruits	**5.59**	**0.12** [a]	3.92	0.09 [b]	2.12	0.09 [c]	<.0001
Meat and its products	9.43	0.15 [b]	6.53	0.11 [c]	**18.05**	**0.38** [a]	<.0001
Eggs	**2.15**	**0.05** [a]	1.86	0.04 [b]	1.41	0.05 [c]	<.0001
Fish and shelfish	3.31	0.08 [b]	3.83	0.07 [a]	3.84	0.13 [a]	<.0001
Seaweeds	0.09	0.00 [b]	**0.12**	**0.00** [a]	0.07	0.00 [c]	<.0001
Milk and dairy products	**5.34**	**0.13** [a]	2.73	0.07 [b]	1.71	0.08 [c]	<.0001
Oils	**3.98**	**0.06** [a]	3.14	0.04 [c]	3.41	0.08 [b]	<.0001
Soda	**1.27**	**0.06** [a]	0.55	0.03 [c]	0.71	0.05 [b]	<.0001
SSBs	3.15	0.07 [a]	3.28	0.06 [a]	2.32	0.08 [b]	<.0001
Non sugar beverage	**0.20**	**0.02** [a]	0.14	0.01 [b]	0.11	0.02 [b]	0.0046
Alcohol	1.07	0.05 [c]	1.23	0.05 [b]	**24.22**	**0.34** [a]	<.0001
Seasonings	3.50	0.07 [a]	2.81	0.04 [b]	3.42	0.08 [a]	<.0001
Processed food	0.03	0.01 [a]	0.00	0.00 [b]	0.02	0.01 [ab]	0.0277
Ect	0.07	0.01 [a]	0.04	0.01 [b]	0.06	0.01 [ab]	0.0252

[1] Values are Mean ± SE. Data were weighted to represent adults aged 19–64 y from KNHANES 2010–2014.

[2]Dietary patterns minus fast food were determined by cluster analysis using intake (kcal/day) and excluding any fast food items consumed.

[3]Statistically different among groups by ANOVA and Duncan's multiple range test.(P < 0.05).
